# Associations between TNF-α-308A/G Polymorphism and Susceptibility with Dermatomyositis: A Meta-Analysis

**DOI:** 10.1371/journal.pone.0102841

**Published:** 2014-08-07

**Authors:** Si Chen, Qian Wang, Ziyan Wu, Qingjun Wu, Ping Li, Yuan Li, Jing Li, Chuiwen Deng, Chanyuan Wu, Lei Gao, Fengchun Zhang, Yongzhe Li

**Affiliations:** 1 Department of Rheumatology and Clinical Immunology, Peking Union Medical College Hospital, Chinese Academy of Medical Sciences & Peking Union Medical College, Key Laboratory of Rheumatology and Clinical Immunology, Ministry of Education, Beijing, China; 2 Key Laboratory of Mental Health, Institute of Psychology, Chinese Academy of Sciences, Beijing, China; Sanjay Gandhi Medical Institute, India

## Abstract

**Background:**

Some surveys had inspected the effects of the tumor necrosis factor-α (TNF-α)-308A/G polymorphism on susceptibility to dermatomyositis (DM), and showed mixed results. To briefly review these consequences, a comprehensive meta-analysis was carried out to estimate the relationship between them much more accurately.

**Methods:**

Relevant documents dated to February 2014 were acquired from the PUBMED, MEDLINE, and EMBASE databases. The number of the genotypes and/or alleles for the TNF-α-308A/G in the DM and control subjects was extracted and statistical analysis was conducted using STATA 11.2 software. Summary odds ratios (ORs) with their 95% confidence intervals (95% CIs) were used to calculate the risk of DM with TNF-α-308A/G. Stratified analysis based on ethnicity and control population source was also performed.

**Results:**

555 patients with DM and 1005 controls from eight published investigations were finally involved in this meta-analysis. Combined analysis revealed that the overall ORs for the TNF-α-308A allele were 2.041 (95% CIs 1.528–2.725, P<0.0001) in DM. Stratification by ethnicity indicated the TNF-α-308A allele polymorphism was found to be significantly associated with DM in Europeans (OR = 1.977, 95% CI 1.413–2.765, P<0.0001). The only study conducted on TNF-α-308A/G polymorphism in Asians could not be used in ethnicity-stratified meta-analysis. Meta-analysis of the AA+AG vs. GG (dominant model) and AA vs. GG (additive model) of this polymorphism revealed a significant association with DM in overall populations and Europeans.

**Conclusions:**

Our meta-analysis demonstrated that the TNF-α-308A/G polymorphism in the TNF gene might contribute to DM susceptibility, especially in European population. However, further studies with large sample sizes and among different ethnicity populations should be required to verify the association.

## Introduction

Idiopathic inflammatory myopathies (IIM) are a heterogeneous group of diseases that affect skeletal muscles. Dermatomyositis (DM) is the most common subtype. Its clinical features are muscle weakness;muscle biopsies which typically show inflammatory cell infiltrates, and specific cutaneous involvement. Although the aetiology of the DM is unclear, genetic factors are thought to contribute to the pathogenesis of DM [1]–[2]. The idiopathic inflammatory myopathies are comparatively scarce, with probable prevalence of 10 to 60 cases per million populations, and this has obstructed progress in genetic studies [3]. However, a few surveys have chiefly focused on the tumor necrosis factor-α (TNF-α) gene polymorphism and DM risk.

TNF-α is a potential pro-inflammatory cytokine that plays a prominent role in inflammatory and immune responses, including those observed in DM [4]. TNF-α is mainly produced by activated macrophages, but also by activated monocytes, neutrophils, T cells and NK cells. The TNF gene is located on chromosome 6, within the class III region of the human lymphocyte antigen (HLA) [5], and some single-nucleotide polymorphisms have been recognized in its promoter [6], such as −308A/G, −238G/A, +489G/A, −1031T/C, −863C/A, −857C/T, which can regulate the transcription and production of TNF-α. Of these, the G-to-A substitution in the promoter at the position –308 has been mostly studied in the TNF gene [7]. It is not clear whether the TNF-α-308A/G polymorphism has operational significance, but it’s believed that the TNF-α-308A/G polymorphism may have a little but significant effect, with the TNF-α- A allele combined with larger levels of TNF-α transcription [6]–[8]. However, the published consequences are inconsistent [9].

Previous studies had examined the potential association between the TNF-α -308A/G polymorphism and the susceptibility to DM [10]–[17]. However, these surveys were inconclusive and kept on contradictory, due to majority studies only enclosed a small sample size, and each of them might have inadequate power to elucidate a positive association and lack the evidence to illustrate an absence of association. Furthermore, the low statistical powers of individual studies could explain the contradictory published results. On the other hand, meta-analysis is a powerful means to synthesize information from varied investigations on the same issue. Hence, we performed this meta-analysis to check whether the TNF-α-308A/G polymorphism contributed to the susceptibility of DM. Based on our knowledge, this was the first meta-analysis of the association between TNF-α-promoter −308A/G polymorphism and DM risk.

## Methods

### Literature search

The electronic databases of PUBMED, MEDLINE, and EMBASE were comprehensively searched, with the following terms utilized: “tumor necrosis factor-α” or “tumor necrosis factor-alpha” or “TNF-α” or “TNF-alpha” and “dermatomyositis” or “idiopathic inflammatory myopathies” and “polymorphism” or “genetic”. All documents were updated to February 2014. The language was limited to English. Additional relevant references quoted in searched articles were also selected.

### Inclusion and Exclusion criteria

Studies meeting the following criteria were included: (1) case–control studies on the association between TNF-α polymorphisms and DM risk; (2) comprised genotype data; (3) sufficient data for evaluating OR with 95% CI.

Studies were excluded if they satisfied the following criteria: (1) studies in which genotype frequencies or alleles could not be ascertained; (2) studies in which family members had been studied; (3) reviews or abstracts; (4) animal studies. For the overlapping studies, only the one with the largest sample size was included in our study.

### Data extraction

Data was extracted from all selected studies by two independent investigators (SC and QW). Inter-researcher disagreements were resolved by consensus or by a third investigator (YL). The following data was collected from each selected study: author, publication year, ethnicity of the subject population, age of population, study-design (sources of controls), demographics, total numbers of patients and controls, and the frequency of the genotypes and alleles of the TNF-α gene-308A/G polymorphism in cases and controls. Authors of the identified studies were contacted via E- mail if further study details were needed.

### Statistical analysis

Under the dominant model (AA+AG vs. GG), recessive model (AA vs. GG+AG), additive model (AA vs. GG) and allele model (A vs. G), we evaluated the strength of associations between TNF-α gene-308A/G polymorphism and the risk of DM by calculating a pooled OR and 95% CI. The statistical significance of OR was ascertained with Z-test, and P<0.05 was deemed to statistically significant. Applying the fixed-effects model or random-effects model depended on the degree of heterogeneity among studies. The Cochran’s Q statistic and the I^2^ statistic were used to assess whether or not heterogeneity existed among the studies included in this meta-analysis. P>0.10 in Q-test indicated lack of heterogeneity among studies, so that the combined OR evaluated of each investigation was calculated by the fixed-effects model. Otherwise, we used the random-effects model. The I^2^-statistic was also calculated to evaluate heterogeneity, with I^2^<25% considered as low heterogeneity, 25%–50% as moderate, and >50% as degree of inconsistency [18]–[19]. Subgroup analysis was conducted with respect to ethnicity. Sensitivity analysis was carried out by successively excluding the low quality studies to assess the stability of the outcomes [20]. The χ2 test was applied to appraise Hardy-Weinberg equilibrium (HWE) in the controls [21]. The potential publication bias was evaluated with the Begg’s funnel plot [22]. Statistical analysis was done using STATA 11.2 software (Stata Corporation, College Station, TX, USA). The powers of each study were computed as the probabilities of detecting associations between the TNF-α polymorphism and DM at the 0.05 level of significance, assuming an OR of 1.5 (small effect size). The power analysis was performed using the statistical program G*Power (http://www.psycho.uni-duesseldorf.de/app/projects/gpower).

## Results

### Studies and populations characteristics

**Figure 1:**
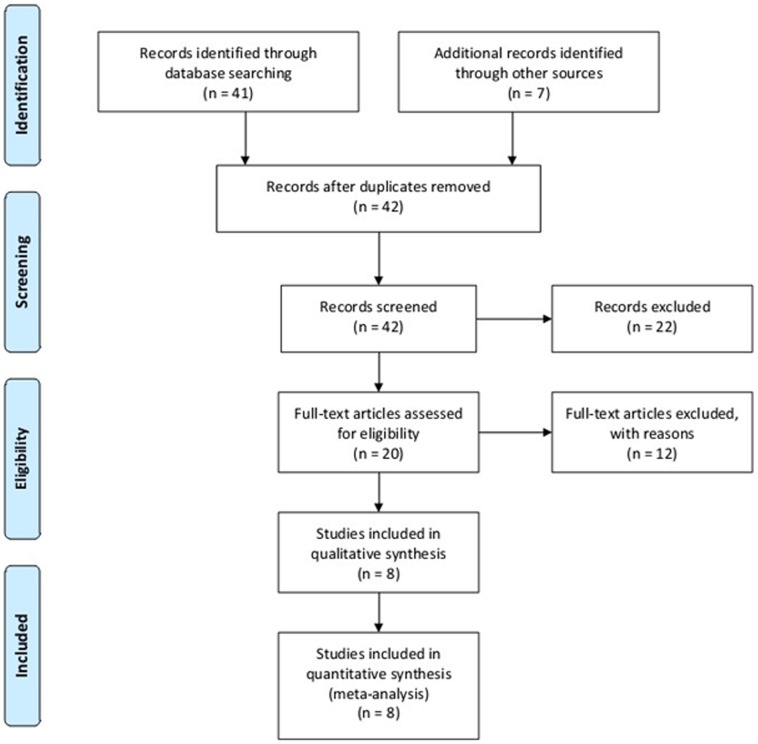
Flow diagram of included/excluded studies.

Using electronic and manual searches identified forty-eight potentially relevant articles and twenty of these were selected for full-text review based on title and abstract details [10]–[17], [23]–[34]. Twelve studies were excluded because they contained no extractable data, other polymorphism data or review [23]–[34]. Finally, a total of eight published documents met our inclusion criteria [10]–[17](Fig. 1). These articles included 555 cases and 1005 controls reporting the relationship between TNF-α-308A/G gene polymorphism and the susceptibility to DM. When stratified by ethnicity, seven essays involving Europeans included 506 patients and 853 controls, and the only article was respected to Asian population containing 49 cases and 152 controls. Thus, the ethnicity-specific meta-analysis was restrained to Europeans. Eight articles had sufficient information to extract the numbers of allele A and allele G in both DM cases and controls. Selected characteristics of each study were summarized in Table 1. The statistical powers of these studies ranged from 21.0 to 83.0%, and only one study had a power of more than 80% (Table 1).

**Table 1:** Characteristics of the studies and populations included in the meta-analysis.

Author(*Ref*)	Year	Countries (Ethnicity)	Age of population	SOC	Numbers		Case			Control			Association P value	P_HWE_	*Power (%)
					Case	Control	AA	AG	GG	AA	AG	GG			
Hassan [10]	2004	Sweden (E)	Adult	HB	29	65	2	15	12	4	18	43	0.016	0.278	27.9
Dourmishev [11]	2012	Bulgarian (E)	Adult	PB	27	63	1	3	23	2	13	48	NS	0.357	26.9
Pachman [12]	2000	Chicago (E)	Juvenile	PB	37	29	5	13	19	1	4	24	0.009	0.167	21.0
Werth [13]	2002	USA (E)	Adult	PB	50	239	2	20	25	3	59	161	<0.03	0.350	67.2
Hristova [14]	2012	Bulgarian (E)	Adult	PB	33	77	1	4	28	1	16	60	NS	0.954	31.7
Mamyrova [15]	2008	USA (E)	Juvenile	PB	221	203	8	132	81	4	59	140	0.0002	0.436	83.0
Liu [16]	2007	China (A)	Adult	PB	49	152	1	17	31	1	27	124	0.009	0.719	51.8
Chinoy [17]	2007	UK (E)	Adult	PB	109	177	5	55	48	2	42	112	0.0001	0.376	66.8

Author: first author’s name, *Ref:* reference; Year: Publication year; USA: United States of America, UK: United Kingdom, E: European, A: Asian; SOC: source of control, HB: hospital-based, PB: population-based; NS no significant; P_HWE:_ P value of Hardy-Weinberg equilibrium, chi-square test; *Power calculations assume α = 0.05, OR = 1.5.

### Frequency of the A allele of the TNF-a −308 A/G polymorphism by ethnicity

The average prevalence of the A allele of the TNF-α-308A/G polymorphism was 23.9% in the control groups. Europeans had a higher A allele frequency than Asian populations (14.4%). Among healthy controls, the frequency of the TNF-α-308A allele in the Asians was 9.5% (Table 2).

**Table 2:** Prevalence of the A allele of the TNF-α gene-308 A/G polymorphism.

Population	Number of studies	Numbers		A allele(%)	
		Case	Control	Case	Control
European	7	506	853	28.7	14.4
Asian	1	49	152	19.4	9.5
Overall	8	555	1005	48.1	23.9

### TNF-α-308A/G polymorphism in meta-analysis

**Figure 2:**
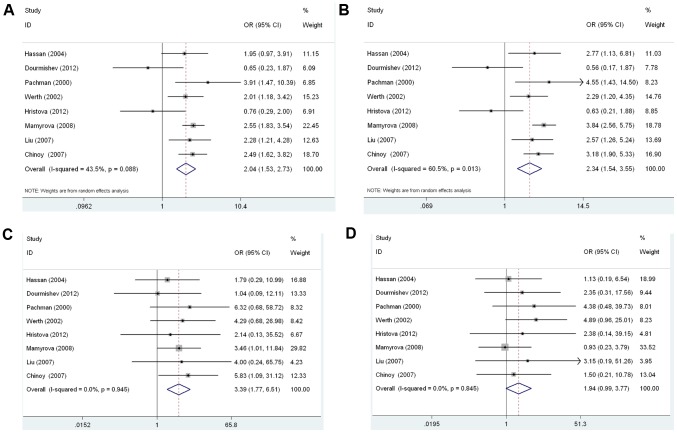
Forest plot of the susceptibility of DM associated with TNF-α-308 gene polymorphism. A: A vs. G, Forest plot; B: AA+AG vs. GG, Forest plot, C: AA vs. GG, D: AA vs. GG+AG.

All eight studies were combined into the meta-analysis. Table 3 provided the summary of the meta-analysis outcomes regarding to the relationship between the TNF-α gene-308A/G polymorphism and DM risk. In overall analysis, there was no evidence of heterogeneity under the additive model (AA vs. GG: I^2^ = 0.0%, P_heterogeneity_ = 0.945), and the recessive model (AA vs. GG+AG: I^2^ = 0.0%, P_heterogeneity_ = 0.845). Therefore, these two genetic models used fixed-effects model. However, the allele model (A allele vs. G allele: I^2^ = 43.5%, P_heterogeneity_ = 0.088), and the dominant model (AA+AG vs. GG: I^2^ = 60.5%, P_heterogeneity_ = 0.013) had apparently between-study heterogeneity. Thus, a random-effects model was utilized for these two genetic models. Significantly increased DM risk was found for A allele vs. G allele (OR = 2.041, 95% CI 1.528–2.725, P<0.0001, Fig. 2A), for AA+AG vs. GG (OR = 2.339, 95% CI 1.544–3.545, P<0.0001, Fig. 2B), for AA vs. GG (OR = 3.391, 95% CI 1.767–6.506, P<0.0001, Fig. 2C) (Table 3). However, not significantly increased risk was found for AA vs. GG+AG (OR = 1.936, 95% CI 0.995–3.767, P = 0.052, Fig. 2D) (Table 3).

**Table 3:** Meta-analysis of the association between the TNF-α-308 A/G polymorphism and DM.

Polymorphism	Population	Number or studies	Test of association			Test of heterogeneity		
			OR	95% CI	P value	Model	P value	I^2^
A versus G allele	Overall	8	2.041	1.528–2.725	<0.0001	R	0.088	43.5
	European	7	1.977	1.413–2.765	<0.0001	R	0.054	51.5
	Asian	1	2.281	1.214–4.283	0.010	NA	NA	NA
AA+AG versus GG (dominant)	Overall	8	2.339	1.544–3.545	<0.0001	R	0.013	60.5
	European	7	2.261	1.393–3.669	0.001	R	0.007	66
	Asian	1	2.571	1.263–5.235	0.009	NA	NA	NA
AA versus GG+AG (recessive)	Overall	8	1.936	0.995–3.767	0.052	F	0.845	0
	European	7	1.886	0.951–3.742	0.070	F	0.772	0
	Asian	1	3.146	0.193–51.257	0.421	NA	NA	NA
AA versus GG	Overall	8	3.391	1.767–6.506	<0.0001	F	0.945	0
	European	7	3.364	1.722–6.569	<0.0001	F	0.897	0
	Asian	1	4.000	0.243–65.753	0.332	NA	NA	NA

OR odds ratio; CI confidence interval; F fixed effects model; R random effects model; NA not available.

**Figure 3:**
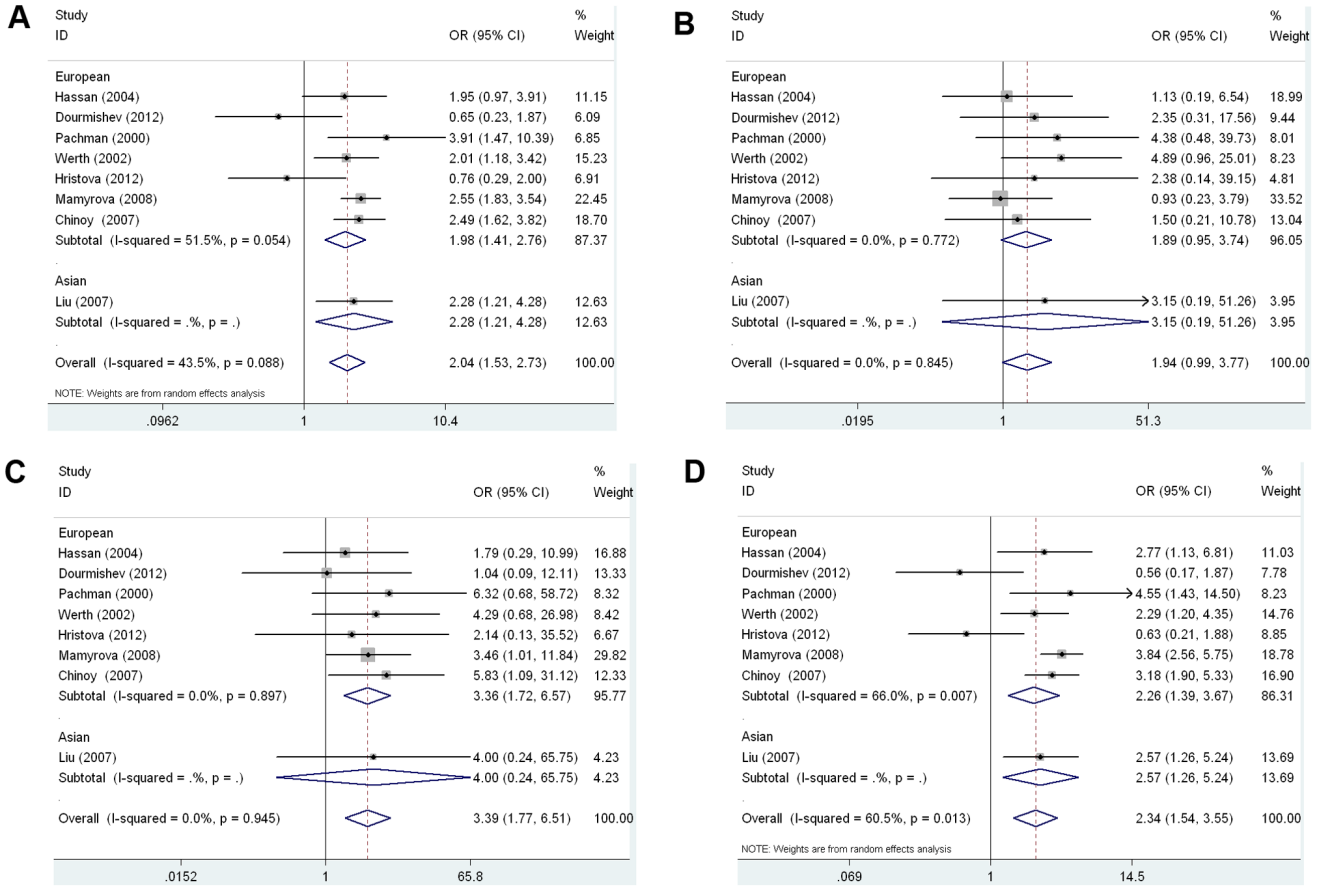
Forest plot of the susceptibility of DM associated with TNF-α-308 gene polymorphism under stratification. A: A vs. G, Forest plot; B: AA+AG vs. GG, Forest plot, C: AA vs. GG, D: AA vs. GG+AG.

Then stratified analysis was executed to assess the potential ethnic differences. All the genetic models of the Europeans were similar to the overall populations. Significantly increased DM risk was also found for A allele vs. G allele (OR = 1.977, 95% CI 1.413–2.765, P<0.0001, Fig. 3A), for AA+AG vs. GG (OR = 2.261, 95% CI 1.393–3.669, P = 0.001, Fig. 3B), for AA vs. GG (OR = 3.364, 95% CI 1.722–6.569, P<0.0001, Fig. 3C) (Table 3). Similarly, the recessive model (AA vs. GG+AG) showed no statistical significance (OR = 1.886, 95% CI 0.951–3.742, P = 0.070, Fig. 3D) (Table 3). However, the only study performed on the TNF-α-308A/G polymorphism in Asians was worthless, because it did not meet the significance of ethnicity-stratified meta-analysis (Table 3).

### Sensitivity Analysis and Publication Bias

**Figure 4:**
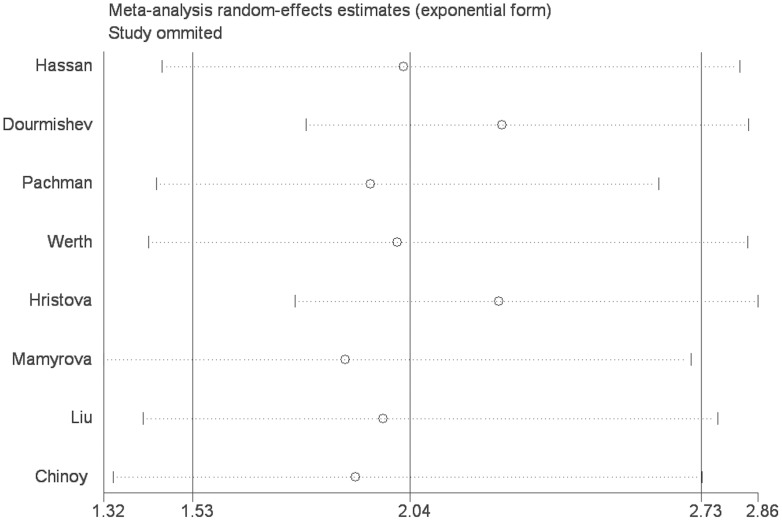
The results of sensitivity analysis from random-effects estimates. (A allele vs. G allele).

To further reinforce our conclusions, the sensitivity analysis was performed by consecutively excluding individual studies. For TNF-α-308A/G polymorphism, the corresponding summary ORs were not changed significantly, indicating that our results were statistically robust (detailed data not shown) (Fig. 4).

**Figure 5:**
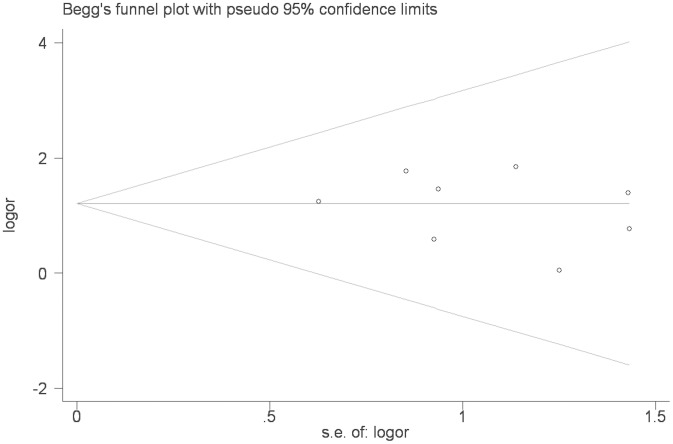
Begg’s funnel plot for publication bias test (AA vs. GG).

To examine publication bias, Begg’s funnel plot and Egger’s test were performed for the association between TNF-α-308A/G polymorphism and DM risk. The shape of the funnel plots showed almost symmetrical. The Egger’s test and Begg’s test indicated that there was no evidence of publication bias (Egger’s test P = 0.560; Begg’s test P = 0.536 for AA vs GG, Fig. 5).

## Discussion

Candidate gene studies need large samples to accomplish sufficient statistical significance and reproducible results [35]. DM is a rare disease. Data on incidence and prevalence in DM between ethnicities were different and conflicting. DM had incidence and prevalence rates of 10 to 60 cases per million populations in different ethnic groups (Europe Continent, America Continent, Asia Continent, et al.)[36]–[41]. These figures were evaluated; the true incidence and prevalence were difficult to ascertain because of the rarity of the disease and the lack of consistent use of diagnostic criteria. Nonetheless, some studies on association between TNF-α–308 polymorphism and DM risk had used comparatively small samples [10]–[14], [16], and the results remained conflicting, but not surprising. Therefore, meta-analysis is imperative to ensure adequate statistical power. We knew that our study was the first meta-analysis assessing the association between TNF-α-308A/G polymorphism and DM risk.

In this meta-analysis, eight case-control articles about the relationship between TNF-α-308A/G gene polymorphism and the susceptibility to DM were selected. These studies included 555 cases and 1005 controls, and seven researches regarding Europeans contained 506 patients and 853 controls, and the only article relating with Asians included 49 cases and 152 controls. The current meta-analysis implied a conspicuously significant relationship between TNF-α-308A/G gene polymorphism and DM risk under the allele model (A allele vs. G allele, P<0.0001), dominant genetic model (AA+AG vs. GG, P<0.0001) and additive model (AA vs. GG, P<0.0001). In addition, Stratified analysis based on ethnicity was performed. Interestingly, we also found apparent association between TNF-α-308A/G gene polymorphism and Europeans for A allele vs. G allele (P<0.0001), for AA+AG vs. GG (P = 0.001), for AA vs. GG (P<0.0001). The only study performed on the TNF-α-308A/G polymorphism in Asians could not be used in ethnicity-stratified meta-analysis. Therefore, more studies were needed to conduct in Asian population. European population showed a higher frequency of the TNF-α-308A allele (14.4%), while Asians showed a lower frequency of the A allele (9.5%). Thereby, the present study indicated TNF-α-308A allele might increase DM risk.

TNF-α is an inducible cytokine, which has an expansive range of pro-inflammatory and immune-stimulatory response. TNF-α gene is located within the HLA class III region in chromosome 6p21.3 [42]. There are several polymorphisms in the promoter region of the TNF gene (−308A/G, −238G/A, +489G/A, −1031T/C, −863C/A, −857C/T) [43]. Some studies had investigated the relationship between TNF-α-238A/G, +489 A/G, −1031T/C, −863C/A, −857C/T polymorphisms and the susceptibility to DM [11], [14]–[15], [17], we also summarized the characteristics of studies and populations on the association between other TNF polymorphisms and DM risk (The details of the characteristics were provided in Table S1, S2A, S2B, S2C, S2D in File S1). And we did meta-analysis on the association between the TNF-α-238A/G, +489 A/G, −1031T/C, −863C/A, −857C/T polymorphisms and the susceptibility to DM (The details of the results were provided in Table S3 in File S1). As the number of studies and subjects between the TNF-α-238A/G, +489 A/G, −1031T/C, −863C/A, −857C/T polymorphisms and DM risk were very small, we only did the analysis in the supplementary material. Only under the allele model (A allele vs. G allele: P<0.0001), and the dominant model (AA+AG vs. GG: P<0.0001), meta-analysis suggested apparently significant on the association between TNF-α-238A/G polymorphism and DM risk; and only under the dominant model (CC+CT vs. TT: P = 0.039), meta-analysis suggested apparently significant on the association between TNF-α-1031T/C polymorphism and DM risk. We failed to get significant and meaningful results in the meta-analysis on the association between the TNF-α+489A/G −857C/T, −863C/A polymorphisms polymorphism and the susceptibility to DM. Therefore, more studies on the association between TNF-α**-**238A/G, +489 A/G, −1031T/C, −863C/A, −857C/T polymorphisms and DM risk were needed to acquire dependable consequence. Hristova et al. [14] indicated that Interleukin-10 (IL-10)-3575T/A polymorphism showed association with DM, and Mamyrova et al. [15] demonstrated that two polymorphisms of Interleukin-1 (IL-1) had associated with DM, but the relationship between these polymorphisms and DM risk didn’t meet the inclusion criteria of meta-analysis. TNF-α-308A/G polymorphism is the most common polymorphism in the promoter. This polymorphism could influence cytokine production [44]–[46], and the TNF-α promoter-308A/G polymorphism had been reported to be associated with several autoimmune disorders [47]–[48]. TNF-α-308A allele had shown to be a stronger transcriptional activator than the common TNF-α-308G allele in vitro, and patients with TNF-α-308GA heterozygosity had increased TNF production [44], [49]. Although a possible association of the TNF-α-308A/G polymorphism with risk of DM was reported, and it was still unknown whether there was a significant association between TNF-α-308A/G polymorphism and the susceptibility to DM [10]–[17]. Therefore, we conducted this meta-analysis and our outcomes revealed that the TNF-α-308A/G polymorphism might be a potential risk factor for DM susceptibility, especially for Europeans.

DM is a complement-mediated vascular endothelial cell disease, which is associated with perimysium inflammation and peri-fascicular muscle fiber atrophy. In the pathogenesis of DM, large numbers of helper T-cells are found within the perimysium and peri-vascular, which induce inflammatory infiltrates. The endomysium and perimysium inflammatory cells express high levels of cytokine, while macrophages are the primary origin of the cytokine. The influential role played by cytokines in the DM has been acknowledged for long [50]. In the DM, TNF-α is the most studied cytokine up to now. TNF-α is produced and expressed by muscle fibers and correlates with muscle fiber regeneration [51]. Pathophysiological effects of TNF-α on muscle fiber function may contribute to weakness and fatigue in patients with DM. Psoriasis and lichen planus (LP) are other idiopathic skin diseases. A meta-analysis demonstrated TNF-α gene-308A/G polymorphism was associated with decreased risk of psoriasis [52]. However, other meta-analysis suggested no association was found between this polymorphism and LP risk in combined analyses [53]. DM is a typical idiopathic skin disease, and the relationship between TNF-α-308A/G and DM was contradictory. This meta-analysis indicated that TNF-α-308A/G polymorphism might contribute to the susceptibility of DM.

Several limitations from the following aspects should be considered when explaining those findings. First, the number of studies and the number of subjects in researches selected in this meta-analysis were limited, which might furnish insufficient power to estimate the association between TNF-α-308A/G polymorphism and DM risk. It was possible that some connected published studies or unpublished articles with negative conclusions were lost. Therefore, more studies were needed to acquire a more dependable consequence. Second, our outcome was based on unadjusted estimates. Thus, a more accurate analysis could be conducted if individual information were obtainable to permit adjustment. Third, most of the included publications were performed in Europeans; the only study was carried out in Asian population. In this study, ethnicity-specific meta-analysis data were only available for European population, and therefore, our results were only applicable to European group. So future studies should evaluate other population. Fourth, although no evident publication bias was identified, potential bias might have distorted the result of the meta-analysis. Finally, due to limited or unavailable data, effect prompted by age, gender and other environmental factors could not be investigated.

This was the first meta-analysis to evaluate the relationship between the TNF-α-308A/G polymorphism and DM risk. Despite of the above limitations, this systematic analysis of the association of TNF-α-308A/G gene polymorphism with DM risk was statistically more persuading than any single study. This meta-analysis reached a strong conclusion that the -308A/G polymorphism might be a potential risk factor for DM susceptibility, especially for Europeans. Accordingly, our results supported the fact that the role of TNF-α-308A/G polymorphism played in the pathogenesis of DM. However, in order to better assess the association between TNF-α-308A/G gene polymorphism with the susceptibility to DM, further investigations should conduct with a larger number of worldwide studies in standardized and unbiased ways.

## Supporting Information

File S1 **File includes Tables S1–S3.** Table S1: Characteristics of the studies and populations on the association between other TNF polymorphisms and DM risk. Table S2A: Characteristics of the studies on the association between TNF-1031 T/C polymorphism and DM risk. Table S2B: Characteristics of the studies on the association between TNF-238 A/G, +489 A/G polymorphisms and DM risk. Table S2C: Characteristics of the studies on the association between TNF-857 T/C polymorphism and DM risk. Table S2D: Characteristics of the studies on the association between TNF-863 C/A polymorphism and DM risk. Table S3: Meta-analysis of the association between other TNF polymorphisms and DM risk.
(DOC)

File S2 **PRISMA Checklist.**
(DOC)
