# Supplementary material for: Associations between TNF-α-308A/G Polymorphism and Susceptibility with Dermatomyositis: A Meta-Analysis
Source: PLoS One. 2014 Aug 7;9(8):e102841. doi: 10.1371/journal.pone.0102841 (PMC4125139; doi:10.1371/journal.pone.0102841)
Supplement: File S1 — File includes Tables S1–S3. Table S1: Characteristics of the studies and populations on the association between other TNF polymorphisms and DM risk. Table S2A: Characteristics of the studies on the association between TNF-1031 T/C polymorphism and DM risk. Table S2B: Characteristics of the studies on the association between TNF-238 A/G, +489 A/G polymorphisms and DM risk. Table S2C: Characteristics of the studies on the association between TNF-857 T/C polymorphism and DM risk. Table S2D: Characteristics of the studies on the association between TNF-863 C/A polymorphism and DM risk. Table S3: Meta-analysis of the association between other TNF polymorphisms and DM risk. (DOC) [file pone.0102841.s001.doc]

**Table S1.** Characteristics of the studies and populations on the association between other TNF polymorphisms and DM risk.

| **Polymorphisms** | **Author(*Ref*)** | **Year** | **Countries (Ethnicity)** | **Age of population** | **SOC** | **Numbers** | |  | **Association P value** | **P_HWE_** | ****Power（%）** |
| --- | --- | --- | --- | --- | --- | --- | --- | --- | --- | --- | --- |
|  |  |  |  |  |  | **Case** | **Control** |  |  |  |  |
| -1031T/C | Dourmishev[1] | 2012 | Bulgarian (E) | Adult | PB | 27 | 63 |  | 0.0876 | 0.186 | 26.9 |
|  | Hristova[2] | 2012 | Bulgarian (E) | Adult | PB | 33 | 77 |  | 0.088 | 0.190 | 31.7 |
|  | Chinoy[4] | 2007 | UK (E) | Adult | PB | 109 | 177 |  | 0.006 | 0.593 | 66.8 |
| -238A/G | Dourmishev[1] | 2012 | Bulgarian (E) | Adult | PB | 27 | 63 |  | NS | 0.357 | 26.9 |
|  | Hristova[2] | 2012 | Bulgarian (E) | Adult | PB | 33 | 77 |  | NS | NA* | 31.7 |
|  | Mamyrova[3] | 2008 | USA (E) | Juvenile | PB | 221 | 203 |  | 0.002 | 0.291 | 83.0 |
| +489A/G | Dourmishev[1] | 2012 | Bulgarian (E) | Adult | PB | 27 | 63 |  | NS | 0.710 | 26.9 |
|  | Hristova[2] | 2012 | Bulgarian (E) | Adult | PB | 33 | 77 |  | 0.09 | 0.873 | 31.7 |
|  | Chinoy[4] | 2007 | UK (E) | Adult | PB | 109 | 177 |  | NS | 0.357 | 66.8 |
| -857C/T | Dourmishev[1] | 2012 | Bulgarian (E) | Adult | PB | 27 | 63 |  | NS | 0.242 | 26.9 |
|  | Hristova[2] | 2012 | Bulgarian (E) | Adult | PB | 33 | 77 |  | 0.09 | 0.873 | 31.7 |
| -863C/A | Dourmishev[1] | 2012 | Bulgarian (E) | Adult | PB | 27 | 63 |  | NS | 0.186 | 26.9 |
|  | Hristova[2] | 2012 | Bulgarian (E) | Adult | PB | 33 | 77 |  | NS | 0.166 | 31.7 |

Author: first author’s name, *Ref:* reference; Year: Publication year; USA: United States of America, UK: United Kingdom, E: European, A: Asian; SOC: source of control, HB: hospital-based, PB: population-based; P_HWE_: P value of Hardy-Weinberg equilibrium, chi-square test; * Hristova’s result demonstrated that TNF-238 only appeared GG genotype in case and control population. We failed to calculate its HWE frequency; **Power calculations assume α = 0.05, OR = 1.5.

**Table S2A.** Characteristics of the studies on the association between TNF-1031 T/C polymorphism and DM risk.

| **Author(*Ref*)** | **Numbers** | |  | **Case** | | |  | **Control** | | |
| --- | --- | --- | --- | --- | --- | --- | --- | --- | --- | --- |
|  | **Case** | **Control** |  | **CC** | **TC** | **TT** |  | **CC** | **TC** | **TT** |
| Dourmishev[1] | 27 | 63 |  | 2 | 6 | 19 |  | 0 | 18 | 45 |
| Hristova[2] | 33 | 77 |  | 2 | 8 | 23 |  | 0 | 20 | 57 |
| Chinoy[4] | 109 | 177 |  | 0 | 28 | 81 |  | 12 | 62 | 99 |

**Table S2B.** Characteristics of the studies on the association between TNF-238 A/G, +489 A/G polymorphisms and DM risk.

| **Polymorphisms** | **Author(*Ref*)** | **Numbers** | |  | **Case** | | |  | **Control** | | |
| --- | --- | --- | --- | --- | --- | --- | --- | --- | --- | --- | --- |
|  |  | **Case** | **Control** |  | **AA** | **AG** | **GG** |  | **AA** | **AG** | **GG** |
| -238 A/G | Dourmishev[1] | 27 | 63 |  | 0 | 0 | 27 |  | 2 | 13 | 48 |
|  | Hristova[2]* | 33 | 77 |  | 0 | 0 | 33 |  | 0 | 0 | 77 |
|  | Mamyrova[3] | 221 | 203 |  | 2 | 7 | 197 |  | 0 | 28 | 175 |
| +489 A/G | Dourmishev[1] | 27 | 63 |  | 1 | 9 | 17 |  | 1 | 10 | 16 |
|  | Hristova[2] | 33 | 77 |  | 1 | 10 | 22 |  | 6 | 32 | 39 |
|  | Chinoy[4] | 109 | 177 |  | 0 | 11 | 95 |  | 0 | 22 | 142 |

*This research’s result demonstrated that TNF-238 only appeared GG genotype in case and control population. We failed to calculate its HWE frequency. And when we did meta-analysis on the association between TNF-238 A/G polymorphism and DM risk, this research was excluded.

**Table S2C.** Characteristics of the studies on the association between TNF-857 T/C polymorphism and DM risk.

| **Author(*Ref*)** | **Numbers** | |  | **Case** | | |  | **Control** | | |
| --- | --- | --- | --- | --- | --- | --- | --- | --- | --- | --- |
|  | **Case** | **Control** |  | **TT** | **TC** | **CC** |  | **TT** | **TC** | **CC** |
| Dourmishev[1] | 27 | 63 |  | 1 | 9 | 17 |  | 3 | 29 | 31 |
| Hristova[2] | 33 | 77 |  | 1 | 10 | 22 |  | 6 | 32 | 39 |

**Table S2D.** Characteristics of the studies on the association between TNF-863 C/A polymorphism and DM risk.

| **Author(*Ref*)** | **Numbers** | |  | **Case** | | |  | **Control** | | |
| --- | --- | --- | --- | --- | --- | --- | --- | --- | --- | --- |
|  | **Case** | **Control** |  | **AA** | **AC** | **CC** |  | **AA** | **AC** | **CC** |
| Dourmishev[1] | 27 | 63 |  | 0 | 10 | 17 |  | 0 | 18 | 45 |
| Hristova[2] | 33 | 77 |  | 1 | 11 | 21 |  | 0 | 21 | 56 |

**Table S3.** Meta-analysis of the association between other TNF polymorphisms and DM risk.

| **Polymorphisms** | **Comparison** | **Number or studies** | **Test of association** | | |  | **Test of heterogeneity** | | |
| --- | --- | --- | --- | --- | --- | --- | --- | --- | --- |
|  |  |  | **OR** | **95%CI** | **P value** |  | **Model** | **P value** | **I^2^** |
| -1031T/C | C versus T allele | 3 | 0.917 | 0.383-2.194 | 0.846 |  | R | 0.008 | 79.1 |
|  | CC+CT versus TT (dominant) | 3 | 0.650 | 0.432-0.979 | **0.039** |  | F | 0.105 | 55.5 |
|  | CC versus TT+CT (recessive) | 3 | 2.005 | 0.046-87.093 | 0.718 |  | R | 0.008 | 79.1 |
|  | CC versus TT | 3 | 1.838 | 0.038-88.738 | 0.758 |  | R | 0.007 | 80.1 |
| -238A/G* | A versus G allele | 2 | 0.284 | 0.144- 0.558 | **＜0.0001** |  | F | 0.184 | 43.2 |
|  | AA+AG versus GG (dominant) | 2 | 0.227 | 0.108-0.475 | **＜0.0001** |  | F | 0.266 | 19.1 |
|  | AA versus GG+AG (recessive) | 2 | 1.578 | 0.277-9.001 | 0.607 |  | F | 0.274 | 16.3 |
|  | AA versus GG | 3 | 1.301 | 0.233-7.276 | 0.765 |  | F | 0.251 | 24.2 |
| +489A/G | A versus G allele | 3 | 0.695 | 0.444-1.087 | 0.111 |  | F | 0.697 | 0 |
|  | AA+AG versus GG (dominant) | 3 | 0.673 | 0.406-1.114 | 0.124 |  | F | 0.724 | 0 |
|  | AA versus GG+AG (recessive) | 3 | 0.506 | 0.097-2.652 | 0.420 |  | F | 0.581 | 0 |
|  | AA versus GG | 3 | 0.425 | 0.080-2.246 | 0.314 |  | F | 0.525 | 0 |
| -857C/T | T versus C allele | 2 | 0.603 | 0.357-1.019 | 0.059 |  | F | 0.737 | 0 |
|  | TT+TC versus CC (dominant) | 2 | 0.538 | 0.288-1.006 | 0.052 |  | F | 0.870 | 0 |
|  | TT versus CC+TC (recessive) | 2 | 0.502 | 0.105-1.943 | 0.387 |  | F | 0.648 | 0 |
|  | TT versus CC | 2 | 0.400 | 0.082-12.126 | 0.256 |  | F | 0.657 | 0 |
| -863C/A** | A versus C allele | 2 | 1.465 | 0.831-2.582 | 0.186 |  | F | 0.823 | 0 |
|  | AA+AC versus CC (dominant) | 2 | 1.499 | 0.789-2.850 | 0.216 |  | F | 0.957 | 0 |
|  | AA versus CC+AC (recessive) | 1 | 7.154 | 0.284-180.253 | 0.232 |  | NA | NA | NA |
|  | AA versus CC | 1 | 7.884 | 0.309-201.082 | 0.211 |  | NA | NA | NA |

OR odds ratio; CI confidence interval; F fixed effects model; R random effects model; NA not available; * Hristova’s result demonstrated that TNF-238 only appeared GG genotype in case and control population. When we did meta-analysis on the association between TNF-238 A/G polymorphism and DM risk, this research was excluded; ** Dourmishev’s result demonstrated that TNF-863 didn’t appear AA genotype in case and control population. When we did meta-analysis under the recessive model (AA vs. CC+AC) and the additive model (AA vs. CC) on the association between TNF-863 C/A polymorphism and DM risk, this research was excluded.

**References:**

1. Dourmishev L, Kamenarska Z, Hristova M, Dodova R, Kaneva R, et al. (2012) Association of TNF-alpha polymorphisms with adult dermatomyositis and systemic lupus erythematosus in Bulgarian patients. Int J Dermatol 51: 1467-1473.

2. Hristova M, Dourmishev L, Kamenarska Z, Kaneva R, Vinkov A, et al. (2012) Association of tumor necrosis factor alpha (TNF-alpha) and interleukin 10 (IL-10) gene polymorphisms in dermatomyositis patients: a pilot study. Acta Dermatovenerol Croat 20: 148-156.

3. Mamyrova G, O'Hanlon TP, Sillers L, Malley K, James-Newton L, et al. (2008) Cytokine gene polymorphisms as risk and severity factors for juvenile dermatomyositis. Arthritis Rheum 58: 3941-3950.

4. Chinoy H, Salway F, John S, Fertig N, Tait BD, et al. (2007) Tumour necrosis factor-alpha single nucleotide polymorphisms are not independent of HLA class I in UK Caucasians with adult onset idiopathic inflammatory myopathies. Rheumatology (Oxford) 46: 1411-1416.
