# Supplementary material for: Associations between TNF-α-308A/G Polymorphism and Susceptibility with Dermatomyositis: A Meta-Analysis
Source: PLoS One. 2014 Aug 7;9(8):e102841. doi: 10.1371/journal.pone.0102841 (PMC4125139; doi:10.1371/journal.pone.0102841)
Supplement: File S2 — PRISMA Checklist. (DOC) [file pone.0102841.s002.doc]

| **Section/topic** | **#** | **Checklist item** | **Reported on page #** |
| --- | --- | --- | --- |
| **TITLE** | | |  |
| Title | 1 | Associations between TNF-α-308A/G polymorphism and susceptibility with dermatomyositis: a meta- analysis | Tile |
| **ABSTRACT** | | |  |
| Structured summary | 2 | *Background*: Some surveys have inspected the effects of the tumor necrosis factor-α (TNF-α)-308A/G polymorphism on susceptibility to dermatomyositis(DM), and showed mixed results. To briefly review these consequences, a comprehensive meta-analysis was carried out to estimate the relationship between them much more accurately.  *Methods*: Interrelated documents dated to February 2014 were acquired from the PUBMED, MEDLINE, and EMBASE databases. The number of the genotypes and/or alleles for the TNF-α-308A/G in the DM and control subjects was extracted and statistical analysis was conducted using STATA 11.2 software. Summary odds ratios (ORs) with their 95% confidence intervals (95% CIs) were used to calculate the risk of DM with TNF-α-308A/G. Stratified analysis based on ethnicity and control population source was also performed.  *Results*: 555 patients with DM and 1005 controls from eight issued investigations were finally involved in this meta-analysis. Combined analysis revealed that the overall ORs for the TNF-α-308A allele were 2.041 (95% CIs 1.528–2.725, P <0.0001) in DM. Stratification by ethnicity indicated the TNF-α-308A allele polymorphism was found to be significantly associated with DM in Europeans (OR = 1.977, 95 % CI 1.413–2.765, P<0.0001). The only study conducted on TNF-α-308A/G polymorphism in Asians had no significance. Meta-analysis of the AA+AG vs. GG (dominant model) and AA vs. GG (additive model) of this polymorphism revealed a significant association with DM in overall populations and Europeans.  *Conclusions*: Our meta-analysis demonstrates that the TNF-α-308A/G polymorphism in the TNF gene might contribute to DM susceptibility, especially in European population. However, further studies with large sample sizes and among different ethnicity populations should be required to verify the association. | Abstract |
| **INTRODUCTION** | | |  |
| Rationale | 3 | Previous studies had examined the potential association between the TNF-α -308A/G polymorphism and the susceptibility to DM [10-17]. However, these surveys were inconclusive and kept on contradictory, due to majority studies only enclosed a small sample size, and each of them might had inadequate power to elucidate a positive association and lack the evidence to illustrate an absence of association. (References presented in the article) | Introduction |
| Objectives | 4 | Meta-analysis is a powerful means to synthesize information from varied investigations on the same issue. Hence, we performed this meta-analysis to check whether the TNF-α-308A/G polymorphism contributed to the susceptibility of DM. To our mind, this was the first meta-analysis of the association between TNF-α-promoter -308A/G polymorphism and DM risk. | Introduction |
| **METHODS** | | |  |
| Protocol and registration | 5 | A literature search was performed for all studies that evaluating the association between the TNF-α -308A/G polymorphism and the susceptibility to DM. | Methods |
| Eligibility criteria | 6 | Essays meeting the following criteria were included: (1) case–control studies on the association between TNF-α polymorphisms and DM risk; (2) comprised genotype data; (3) sufficient data for evaluating OR with 95%CI. | Methods |
| Information sources | 7 | The electronic databases of PubMed, Medline, Embase were comprehensively searched. | Methods |
| Search | 8 | The following key words were searched: “tumor necrosis factor-α” or “tumor necrosis factor-alpha” or “TNF-α” or “TNF-alpha” and “dermatomyositis” or “idiopathic inflammatory myopathies” and “polymorphism” or “genetic”. All documents were updated to February 2014. Additional connected references quoted in searched articles were also selected. | Methods |
| Study selection | 9 | Two individual investigators (SC & QW) evaluated the references respectively. Decision for inclusion was made on consensus. Essays meeting the following criteria were included: (1) case–control studies on the association between TNF-α polymorphisms and DM risk; (2) comprised genotype data; (3) sufficient data for evaluating OR with 95%CI. | Methods |
| Data collection process | 10 | We collected the full text from internet if possible. We also contacted authors to request full text review or specific data of studies if there was no electronic version of the full text or sufficient data of studies needed for this meta-analysis. | Methods |
| Data items | 11 | Search studies: all studies that evaluating the association between the TNF-α -308A/G polymorphism and the susceptibility to DM. | Methods |
| Risk of bias in individual studies | 12 | The χ2 test was applied to appraise Hardy-Weinberg equilibrium in the controls [21].(References presented in the article) | Methods |
| Summary measures | 13 | Point estimate of risk ratio (RR) and 95% confidence intervals (CI) were calculated. | Methods |
| Synthesis of results | 14 | Under the dominant model (AA+AG vs. GG), recessive model (AA vs. GG+AG), additive model (AA vs. GG) and allele model (A vs. G), we evaluated the strength of associations between TNF-α gene-308A/G polymorphism and the risk of DM by calculating a pooled OR and 95%CI. The statistical significance of OR was ascertained with Z test, and P < 0.05 was deemed to statistically significant. Applying the fixed-effects model or random-effects model depended on the degree of heterogeneity among studies. The Cochran’s Q statistic and the I^2^ statistic were used to assess whether or not heterogeneity existed among the studies included in this meta-analysis. P＞0.10 in Q-test indicated lack of heterogeneity among studies, so that the combined OR evaluated of each investigation was calculated by the fixed-effects model. Otherwise, we used the random-effects model. The I^2^-statistic was also calculated to evaluate heterogeneity, with I^2^<25% considered as low heterogeneity, 25%–50% as moderate, and>50% as degree of inconsistency [18-19]. (References presented in the article) | Methods |

Page 1 of 2

| **Section/topic** | **#** | **Checklist item** | **Reported on page #** |
| --- | --- | --- | --- |
| Risk of bias across studies | 15 | The potential publication bias was evaluated with the Begg’s funnel plot [22]. (References presented in the article) | Methods |
| Additional analyses | 16 | Subgroup analysis was conducted with respect to ethnicity. Sensitivity analysis was carried out by successively excluding the low quality studies to assess the stability of the outcomes [20]. (References presented in the article) | Methods |
| **RESULTS** | | |  |
| Study selection | 17 | All of 48 records were identified for screening. 8 studies including 555 patients were included. The other details are presented in Figure1 (flow diagram). | Results |
| Study characteristics | 18 | Data extracted from the 8 included studies were summarized in Table 1. | Results &Table 1 |
| Risk of bias within studies | 19 | The results were shown in Table 1. | Results &Table 1 |
| Results of individual studies | 20 | RR and 95% CI for each study are presented in Figure 2 and Figure 3. | See Figure2, Figure3 |
| Synthesis of results | 21 | In overall analysis, significantly increased DM risk was found for allele model (A allele vs. G allele: OR=2.041, 95%CI 1.528-2.725, P <0.0001, Fig 2A), for dominant model (AA+AG vs. GG: OR=2.339, 95%CI 1.544-3.545, P <0.0001, Fig 2B), for additive model (AA vs. GG: OR=3.391, 95%CI 1.767-6.506, P <0.0001, Fig 2C) (Table 3). However, not significantly increased risk was found for recessive model (AA vs. GG+AG: OR=1.936, 95%CI 0.995-3.767, P =0.052, Fig 2D) (Table 3). | Results |
| Risk of bias across studies | 22 | The shape of the funnel plots showed almost symmetrical. The Egger’s test and Begg’s test indicated that there was no evidence of publication bias (Egger’s test P = 0.560; Begg’s test P = 0.536 for AA vs GG, Fig 5). | Results |
| Additional analysis | 23 | The sensitivity analysis was performed by consecutively excluding individual studies. For TNF-α-308A/G polymorphism, the corresponding summary ORs were not changed significantly, indicating that our results were statistically robust (detailed data not shown) (Fig 4).  Then stratified analysis was executed to assess the potential ethnic differences. In Europeans, significantly raised DM risk was also found for A allele vs. G allele (OR=1.977, 95%CI 1.413-2.765, P <0.0001, Fig 3A), for AA+AG vs. GG (OR=2.261, 95%CI 1.393-3.669, P=0.001, Fig 3B), for AA vs. GG (OR=3.364, 95%CI 1.722-6.569, P <0.0001, Fig 3C) (Table 3). Similarly, the recessive model (AA vs. GG+AG) showed no statistical significance (OR=1.886, 95%CI 0.951-3.742, P =0.070, Fig 3D) (Table 3). However, the only study performed on the TNF-α-308A/G polymorphism in Asians was of no use, because it did not meet the significance of meta-analysis (Table 3). | Results |
| **DISCUSSION** | | |  |
| Summary of evidence | 24 | The analysis based on the data extracted from included eight case-control articles about the relationship between TNF-α-308A/G gene polymorphism and the susceptibility to DM. This result indicated TNF-α-308A allele might increase DM risk. | Discussion |
| Limitations | 25 | 1. The number of studies and the number of subjects in researches selected in this meta-analysis were limited, which might furnish insufficient power to estimate the association between TNF-α-308A/G polymorphism and DM risk. It was possible that some connected published studies or unpublished articles with negative conclusions were lost. Therefore, more studies were needed to acquire a more dependable consequence. 2. Our outcome was based on unadjusted estimates. Thus, a more accurate analysis could be conducted if individual information were obtainable to permit adjustment. 3. Most of the included publications were performed in Europeans; the only study was carried out in Asian population. In this study, ethnicity-specific meta-analysis data were only available for European population, and therefore, our results were only applicable to European group. So future studies should evaluate other populations. 4. Although no evident publication bias was identified, potential bias might have distorted the result of the meta-analysis. 5. Due to limited or unavailable data, effect prompted by age, gender and other environmental factors could not be investigated. | Discussion |
| Conclusions | 26 | This meta-analysis reached a strong conclusion that the -308A/G polymorphism may be a potential risk factor for DM susceptibility, especially for Europeans. | Discussion |
| **FUNDING** | | |  |
| Funding | 27 | This work was supported by funding from the Research Special Fund for Public Welfare Industry of Health No. 201202004 (to F-C. Z.), and the National Natural Science Foundation of China Grants 81072486, 81172857,81373188 (to Y-Z. L.). | Funding |

*From:*  Moher D, Liberati A, Tetzlaff J, Altman DG, The PRISMA Group (2009). Preferred Reporting Items for Systematic Reviews and Meta-Analyses: The PRISMA Statement. PLoS Med 6(6): e1000097. doi:10.1371/journal.pmed1000097

For more information, visit: **www.prisma-statement.org**.

Page 2 of 2
